# School Nutrition Program; Prevention of Obesity and Fatty Liver in Children

**Published:** 2014-06-29

**Authors:** Gelayol Ardalan, Seyed-Moayed Alavian

**Affiliations:** 1 Ministry of Health and Medical Education; 2 Baqiyatallah Research Center for Gastroenterology and Liver Diseases, Baqiyatallh University of Medical Sciences; 3 Middle East Liver Disease Center, Tehran, Iran

Nonalcoholic fatty liver disease (NAFLD) has become the most common cause of chronic liver disease in the pediatric population in the last few decades^[^^1^^,^^2^^]^. In Western countries, the prevalence of NAFLD is estimated up to 10% of children. In Iran fatty liver was diagnosed by ultrasound in 7.1% of children aged 7-18 years^[^^1^^]^. Increased prevalence of this form of liver disease runs parallel with the dramatic rise in childhood obesity and diabetes mellitus over the past 2 decades. Most of children with NAFLD are low active and obese, and suffer from metabolic impairments (including increased baseline waist circumference, hypertension and high insulin level) and dyslipidemia^[^^3^^]^. There was a strong relationship between NAFLD and the abnormal metabolic variables in children^[^^4^^]^. Higher age, higher fasting insulin, abnormality in total cholesterol, LDL cholesterol and triglycerides is linked with higher risk of NAFLD^[^^1^^]^. Pediatric NAFLD is not a benign condition, and it can be a predictive of cardiovascular disease and diabetes mellitus in adulthood and some times it can progress to severe liver diseases^[^^4^^]^.

 The mechanisms leading to NAFLD in children is the same as in adults and include genetic background, epigenetics and environmental factors (i.e. high caloric intake, daily consumption of junk food and soft drinks, low level of physical activity, and beyond average weight) all of which concur during development and progression of the disease^[^^5^^]^. Obesity is the circle between the risk factors showing that 70% of NAFLD in pediatric group is associated with obesity^[^6^]^. Faghih et al found a tremendous trend toward over-weight and obesity with a 3-fold increase of obesity among adolescent girl students in Ahvaz between 1997 and 2006. The data showed that there was a significant relation between BMI and food habits including number of meals and missing the breakfast. The authors recommend, regarding the harmful ramifications of obesity, to encourage healthy eating patterns and increased physical activity among adolescent girls^[^^7^^]^.

 Diagnosis is established by liver biopsy. Laboratory tests and radiographic findings provide clues to the potential presence of fatty liver disease. Liver enzymes elevation in childhood is a cardio-metabolic risk factor and an additional component of the metabolic syndrome^[^^8^^]^. In all children with abnormal level of liver enzymes, evaluation of NAFLD should be done. To screen children susceptible to NAFLD, it will be very useful to assess waist circumference, fasting blood sugar, fasting insulin and serum lipid profile in regular intervals.

 Nutrition plays essential role in development of NAFLD and it can regress the fatty accumulations in the liver. Lifestyle modification, including slow and steady weight loss, improved dietary habits, and increased daily, aerobic physical activity, remains the first-line approach in treating pediatric fatty liver disease^[^^4^^]^. The ministries of health and education have agreed in 2007 about the instructions for healthy eating in schools. Healthy nutrition bases or markets inside the schools should be established for providing, maintaining and promotion of physical, mental and social activities of the students. The main goals are providing healthy food and avoiding unhealthy food supply for students. Healthy food means providing body’s nutritional needs with safe, adequate, varied and balanced foods. It is a common knowledge that healthy food contains low salt and low-fat with less than 10% of trans-fatty oil. Soft drinks should be eliminated from student’s regimen, mineral water and fresh fruits can replace it.
